# Supplementary material for: Regulatory Dynamics of Cell Differentiation Revealed by True Time Series From Multinucleate Single Cells
Source: Front Genet. 2021 Jan 8;11:612256. doi: 10.3389/fgene.2020.612256 (PMC7820898; doi:10.3389/fgene.2020.612256)
Supplement: Supplementary file 1 [file Data_Sheet_1.ZIP › Supplementary Files & Code/(4) Compute T-invariants/Read_Me_Compute_T-invariants.pdf]

Computation of T-Invariants with Charlie

T-invariants of Petri nets can be computed with Charlie and visualized in Snoopy. Snoopy and Charlie are platform-independent tools that are freely available for download at Monika Heiner's website:

Snoopy download:

https://www-dssz.informatik.tu-cottbus.de/DSSZ/Software/Snoopy

Charlie download:

https://www-dssz.informatik.tu-cottbus.de/DSSZ/Software/Charlie

A short video tutorial which demonstrates the computation of T-invariants with Charlie is available as Movie 1 in the supplementary data to Werthmann, B. and W. Marwan (2017): *J. Phys. D: Appl. Phys.* **50,** 464003

https://iopscience.iop.org/article/10.1088/1361-6463/aa8e2b
