# Supplementary material for: Regulatory Dynamics of Cell Differentiation Revealed by True Time Series From Multinucleate Single Cells
Source: Front Genet. 2021 Jan 8;11:612256. doi: 10.3389/fgene.2020.612256 (PMC7820898; doi:10.3389/fgene.2020.612256)
Supplement: Supplementary file 1 [file Data_Sheet_1.ZIP › Supplementary Files & Code/(0) Read_Me/Read_Me_Supplementary Data and Code.pdf]

Regulatory dynamics of cell differentiation revealed by true time series from multinucleate single cells

Anna Pretschner[1], Sophie Pabel[1], Markus Haas[1], Monika Heiner[2], Wolfgang Marwan[1]*

[1] Magdeburg Centre for Systems Biology and Institute of Biology, Otto von Guericke University, Pfälzer Strasse 5, Magdeburg, Germany

[2] Computer Science Institute, Brandenburg University of Technology Cottbus, Postbox 10 13 44, 03013 Cottbus, Germany

Supplementary Data and Code

This folder contains the complete pipeline for generation and coloring of Petri nets from gene expression data as it was used for creating the figures of the paper, including the data set, the code, appropriate example files, and documentation.

The measurements as described in the paper are stored in the file named:
"anna_zeitreihe_neu01.csv"

To reproduce the computational results, run the code step by step in the following temporal order as indicated by the numbered names of the folders:

(1) Generate Histograms and Evaluate Time Series

(2) Clustering with Simprof Algorithm

(3) Generate Snoopy File with GetPN

(4) Compute T-invariants

(5) Color Snoopy File

To run the R code, simply drag any subfolder named "myR" into the desktop folder of your computer and execute the code under R or RStudio. For demonstration purposes, "myR" contains all files you need at each particular step.

For additional information regarding steps (2) and (3) see:
Rätzel *et al.* (2020): *BioSystems* **189**, 104092

For additional information regarding step (4) see:
Werthmann, B. and W. Marwan (2017): *J. Phys. D: Appl. Phys.* **50,** 464003
