# Supplementary material for: Regulatory Dynamics of Cell Differentiation Revealed by True Time Series From Multinucleate Single Cells
Source: Front Genet. 2021 Jan 8;11:612256. doi: 10.3389/fgene.2020.612256 (PMC7820898; doi:10.3389/fgene.2020.612256)
Supplement: Supplementary file 1 [file Data_Sheet_1.ZIP › Supplementary Files & Code/(3) Generate Snoopy File with GetPN/(1) Read_Me_Get_Petri_Nets.pdf]

Generate Petri Nets as Snoopy Files from Petri Net Description Vectors

Petri nets are generated from Petri net description vectors in ANDL file format as described by Rätzel, V. *et al.* (2020) Biosystems 189, 104092. https://www.sciencedirect.com/science/article/abs/pii/S0303264719304575?via%3Dihub

The software GetPN, together with documentation and example files, has been originally provided as SI_File_Package_3.zip as part of the Supplementary data to Rätzel *et al.* (2020). ANDL files, once generated, are subsequently imported into Snoopy to visually display the Petri nets.

Making ANDL files from Petri net description vectors

To run the Petri net construction program GetPN.exe under DOSBox, do the following:

Copy the provided folder named DOS in your user directory. On my Mac it looks like this:

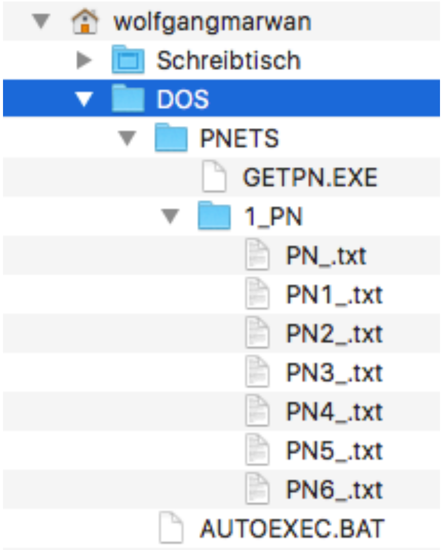

Optionally, remove Autoexec.bat in case you do not want to use it or change it according to your requirements (see below).

PN_.txt, PN1_.txt *etc.* within the 1_PN folder are example files containing Petri net description vectors that are taken as input by GetPN.exe.

Installing DOSBox

Download DOSBox at

https://www.dosbox.com

and install it in the Applications folder of your computer

After starting DOSBox, the following window will pop up:

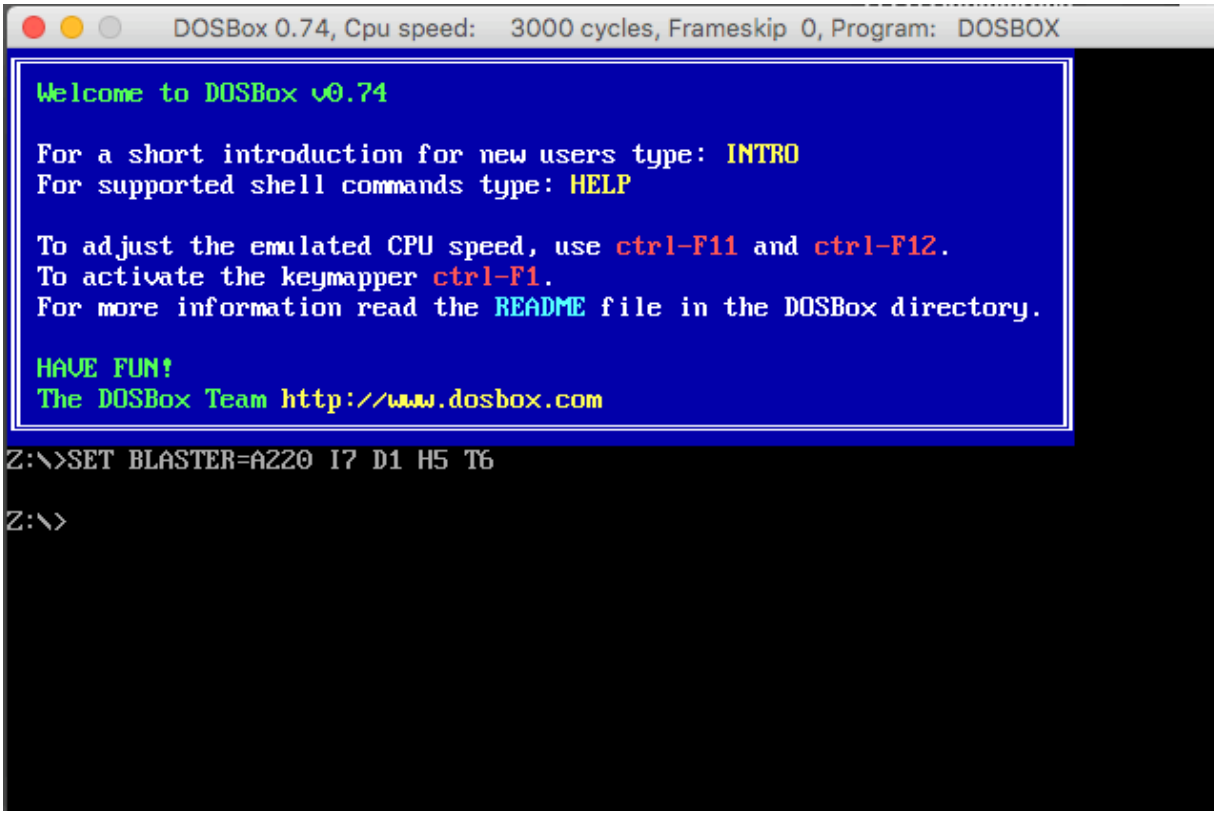

Then mount drive C: with folder DOS as local directory by entering

    mount c ~/dos

on a German keyboard you may type

    mount c >-dos

Then, change to drive c: and change directory to PNets as shown in the screenshot:

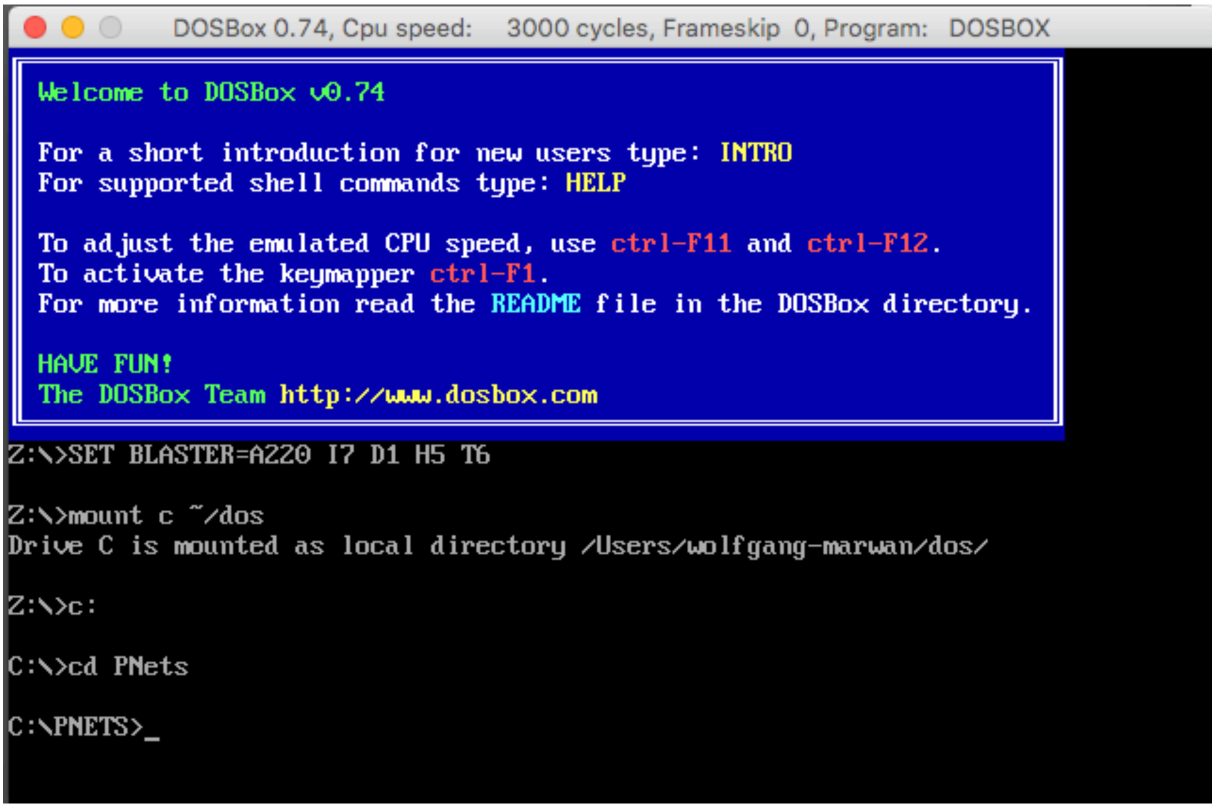

The folder DOS is now set as working directory.

You may alternatively wish to run an autoexec.bat file for convenience just after mounting drive c as local directory by entering

c:autoexec.bat

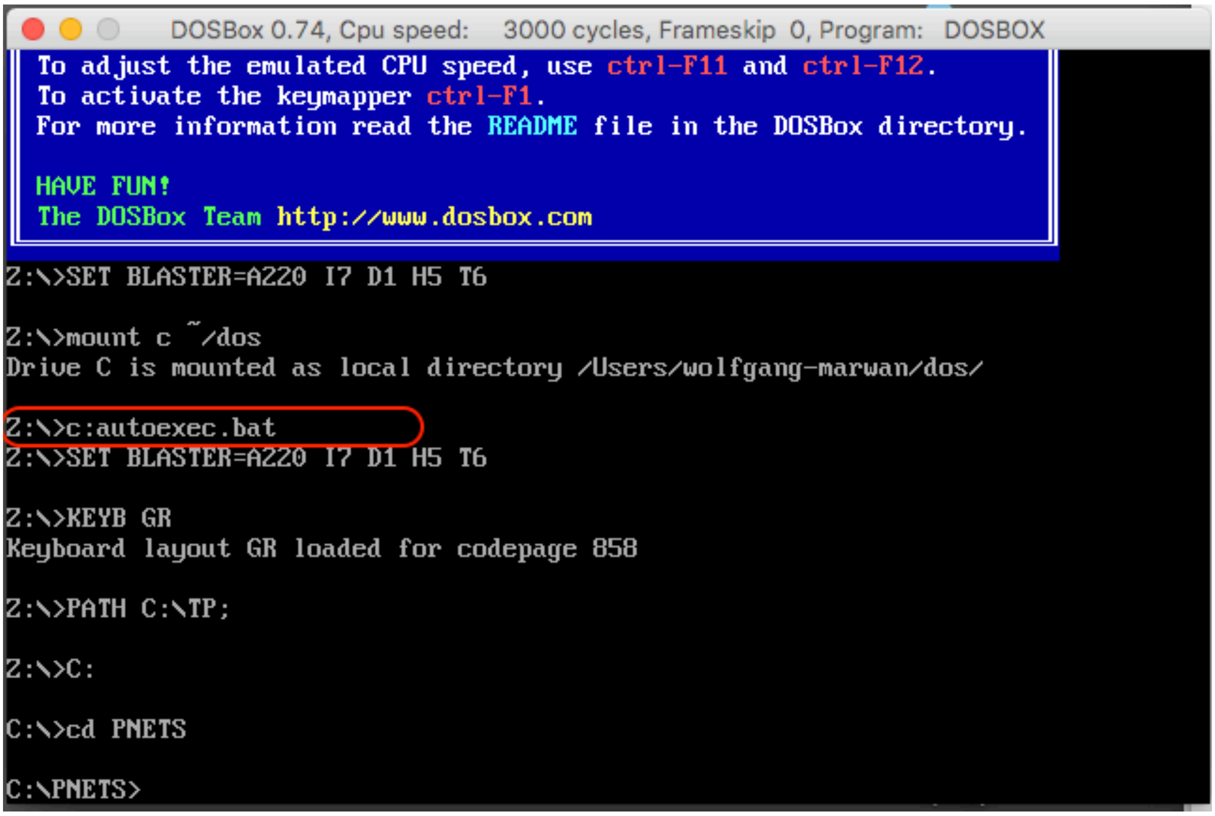

As a template, we have included autoexec.bat file with the following content:

```
SET BLASTER=A220 I7 D1 H5 T6
KEYB GR
PATH C:\TP;
C:
cd PNETS
```

This template can be modified with a text editor.
The command "KEYB GR" switches the keyboard to German. For other country-specific settings see e.g.:
https://www.computerhope.com/keybhlp.htm

The command

    PATH C:\TP;

tells DOS where the TURBO Pascal files can be found (in case they are installed in the folder \TP

Running GetPN

To create ANDL-Files from the Petri net description vector files, simply enter

    GetPN

and press the return button.

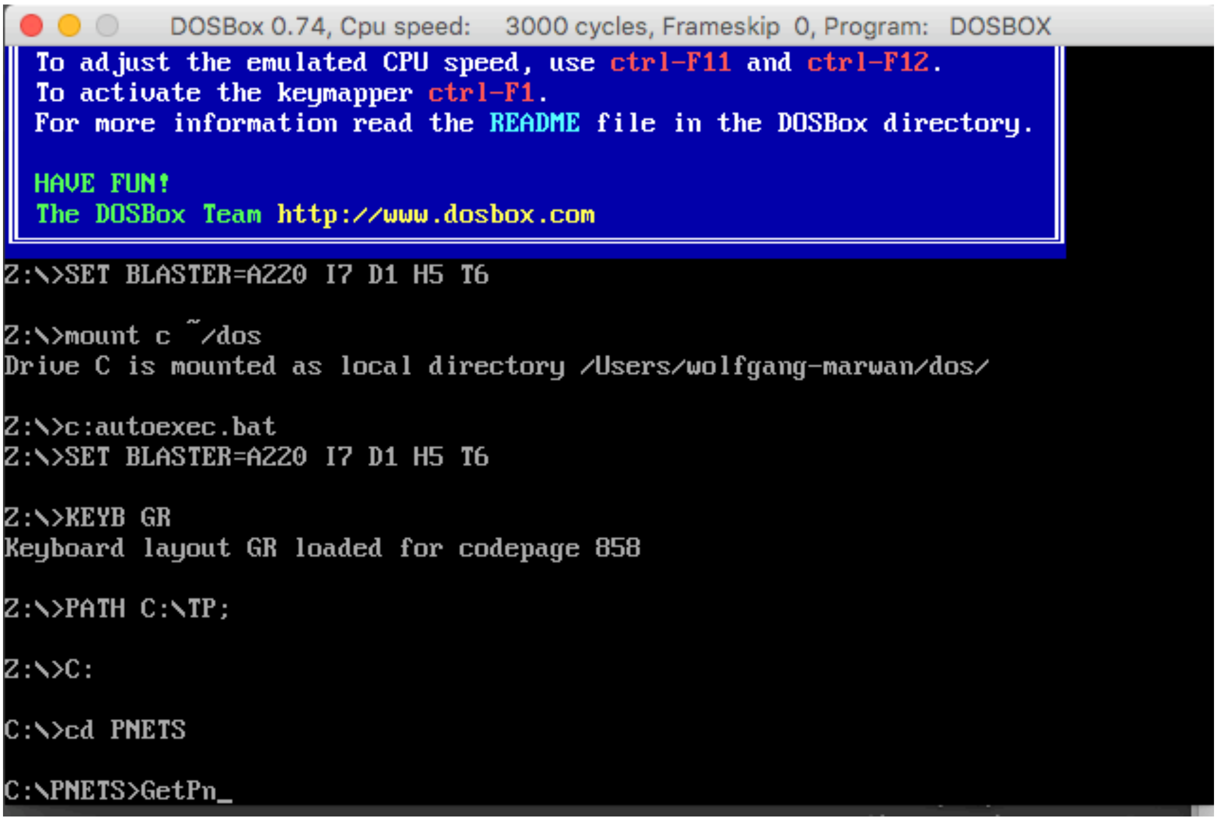

The program will then take you through a dialogue to create the files depending on the options chosen by the user. With PN1_.txt to PN6_.txt, for example, this could look as follows:

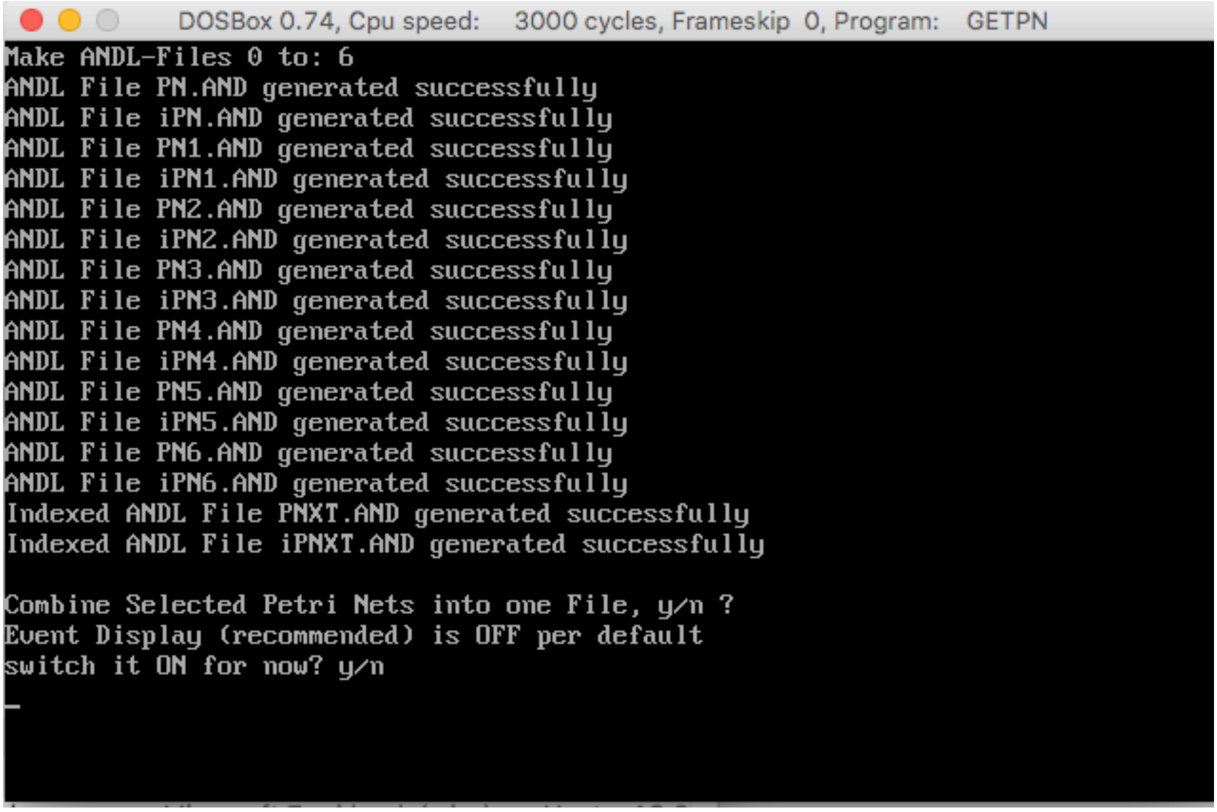

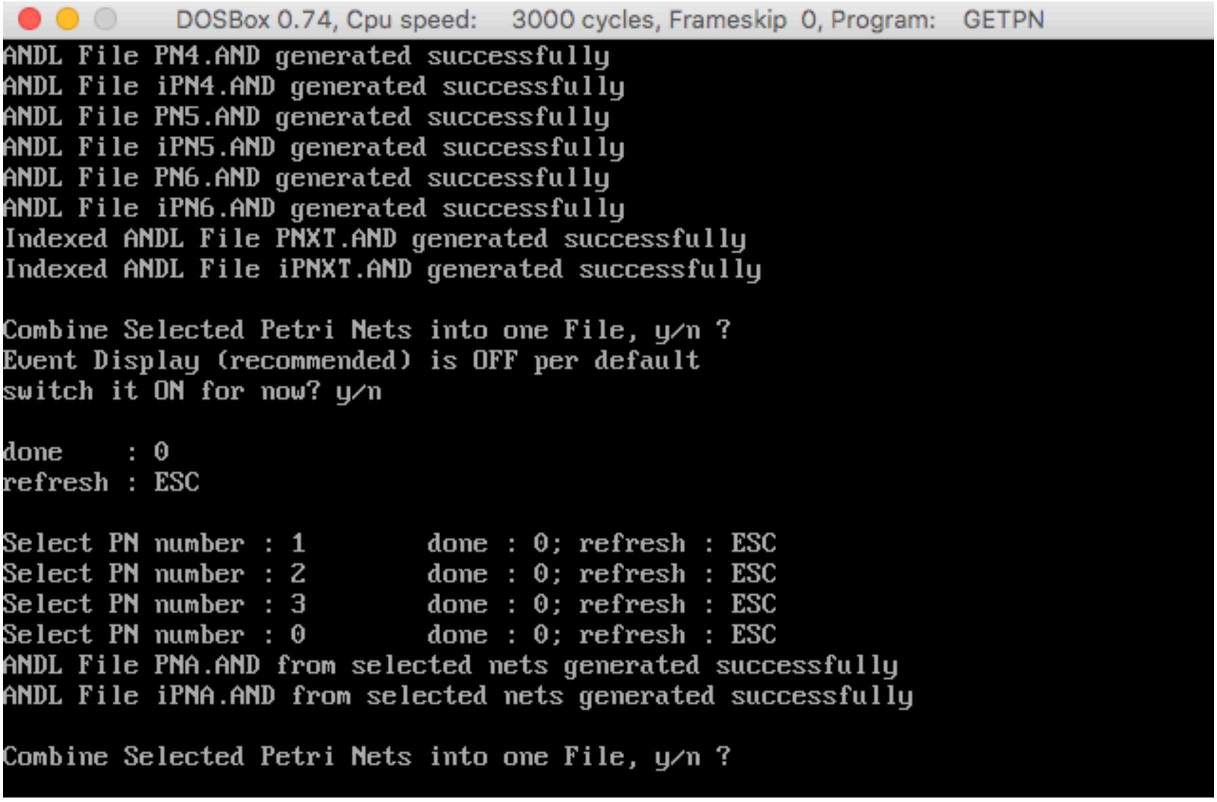

For a description and explanation of file names, refer to the README.TXT file which is created along with the other files.

Working with the TURBO Pascal source code (optional)

The files containing the Turbo Pascal source code

GetPN.PAS

and

MacTP.PAS

can be modified and compiled when TURBO Pascal 5.0 or higher is installed in the folder c:\TP

Renaming the file extensions (essential)

File extensions under DOS support three characters only. Before being imported into Snoopy, the file extensions ".TXT" must be changed to ".andl". In the present study this was done by batch processing under Mac OS X using *Better Rename 9*.
