# Supplementary material for: Regulatory Dynamics of Cell Differentiation Revealed by True Time Series From Multinucleate Single Cells
Source: Front Genet. 2021 Jan 8;11:612256. doi: 10.3389/fgene.2020.612256 (PMC7820898; doi:10.3389/fgene.2020.612256)
Supplement: Supplementary file 2 [file Data_Sheet_2.PDF]

Regulatory dynamics of cell differentiation revealed by true time series from multinucleate single cells

Anna Pretschner[1], Sophie Pabel[1], Markus Haas[1], Monika Heiner[2], Wolfgang Marwan[1]*

[1] Magdeburg Centre for Systems Biology and Institute of Biology, Otto von Guericke University, Pfälzer Strasse 5, Magdeburg, Germany

[2] Computer Science Institute, Brandenburg University of Technology Cottbus, Postbox 10 13 44, 03013 Cottbus, Germany

Supplementary Tables and Figures

SI Table 1. Quantil distributions of the x-fold deviation (x) of a value from the median of values measured in dark controls or far-red stimulated cells. The table quantitatively characterizes the frequency distributions shown in SI Fig. 1B,C.

	**Dark**		**Far-red**	
Percent of values	Quantile (Log2(x))	Quantile (x)	Quantile (Log2(x))	Quantile (x)
1%	-1.055	0.481	-1.040	0.486
5%	-0.593	0.663	-0.609	0.655
25%	-0.186	0.879	-0.185	0.880
50%	0.000	1.000	0.000	1.000
75%	0.181	1.134	0.180	1.133
95%	0.584	1.499	0.622	1.539
99%	1.017	2.024	1.073	2.103

SI Table 2. Genes analysed.

Transcript ID	Gene	Similarity	Organism	UniProtKB	% Query Coverage	E-value
Phypoly_transcript_07700	*anxA*	Annexin VII	*Mus musculus*	Q07076	39.157	1.04E-32
Phypoly_transcript_06541	*ardA*	Actin, plasmodial isoform	*Physarum polycephalum*	P02576	100	0
Phypoly_transcript_11686	*arpA*	Probable basic-leucine zipper transcription factor G	*Dictyostelium discoideum*	Q54RZ9	44.068	1.15E-22
Phypoly_transcript_05813	*cdcA*	Caltractin isoform 1	*Homo sapiens*	P41208	59.756	1.26E-32
Phypoly_transcript_06284	*cudA*	Putative transcriptional regulator cudA	*Dictyostelium discoideum*	O00841	43.478	3.96E-28
Phypoly_transcript_01287	*damA*	DNA damage-binding protein 1	*Oryza sativa Japonica Group*	Q6L4S0	61.892	0
Phypoly_transcript_03245	*dspA*	DNase TatD	*Pantoea vagans*	E1SKR8	28.986	9.63E-31
Phypoly_transcript_06735	*ehdA*	EH domain-containing protein 1	*Bos taurus*	Q5E9R3	41.315	9.57E-130
Phypoly_transcript_16748	*gapA*	ADP-ribosylation factor GTPase-activating protein	*Arabidopsis thaliana*	O82171	35.22	2.61E-24
Phypoly_transcript_17984	*hcpA*	Anti-silencing function protein 1 homolog A	*Bos taurus*	Q2KIG1	52.88	8.27E-64
Phypoly_transcript_18509	*hstA*	Probable histone H2B 4	*Caenorhabditis elegans*	Q27876	59.559	2.41E-37
Phypoly_transcript_16372	*ligA*	Checkpoint protein hus1 homolog	*Dictyostelium discoideum*	Q54NC0	41.241	7.24E-61
Phypoly_transcript_00697	*meiB*	Protein MEI2-like 5	*Arabidopsis thaliana*	Q8VWF5	73.545	6.34E-80
Phypoly_transcript_18969	*nhpA*	Non-histone chromosomal protein 6	*Debaryomyces hansenii*	Q6BRB4	55.224	4.57E-15
Phypoly_transcript_00387	*pakA*	Serine/threonine-protein kinase *pakA*	*Dictyostelium discoideum*	Q55D99	41.085	1.96E-87
Phypoly_transcript_13303	*pcnA*	Proliferating cell nuclear antigen	*Brassica napus*	Q43124	55.484	1.27E-81
Phypoly_transcript_00857	*pikB*	Phosphatidylinositol 3-kinase 2	*Dictyostelium discoideum*	P54674	68.809	0
Phypoly_transcript_01882	*pikC*	Phosphatidylinositol 4-kinase beta	*Sorex araneus*	B3EX61	45.667	6.23E-76
Phypoly_transcript_02833	*pksA*	Serine/threonine-protein kinase phg2	*Dictyostelium discoideum*	Q54QQ1	50.68	1.34E-93
Phypoly_transcript_02552	*pldA*	Phosphatidylinositol-glycan-specific phospholipase D	*Rattus norvegicus*	Q8R2H5	32.237	2.26E-71
Phypoly_transcript_04506	*pldB*	Phosphatidylinositol-glycan-specific phospholipase D	*Homo sapiens*	P80108	28.774	4.91E-49
Phypoly_transcript_02197	*pldC*	Phosphatidylcholine-hydrolyzing phospholipase D1	*Schizosaccharomyces pombe*	Q09706	31.068	7.83E-55
Phypoly_transcript_23026	*pptA*	Probable inactive purple acid phosphatase 29	*Arabidopsis thaliana*	Q9FMK9	46.269	1.10E-09
Phypoly_transcript_08298	*pptB*	Probable protein phosphatase 2C 34	*Oryza sativa Japonica Group*	Q94H98	30.208	1.11E-28
Phypoly_transcript_16094	*psgA*	*Physarum*-specific gene A	*Physarum polycephalum*			
Phypoly_transcript_00670	*pumA*	Pumilio homolog 1	*Arabidopsis thaliana*	Q9ZW07	61.281	1.91E-138
Phypoly_transcript_11692	*pwiA*	Piwi-like protein Ago3	*Bombyx mori*	A9ZSZ2	30.672	8.11E-37
Phypoly_transcript_17606	*ralA*	Circularly permutated Ras protein 1	*Dictyostelium discoideum*	Q75J93	39.161	7.24E-21
Phypoly_transcript_09675	*rasA*	Ras-like GTP-binding protein YPT1	*Phytophthora infestans*	Q01890	43.931	3.66E-43
Phypoly_transcript_12613	*rgsA*	Regulator of G-protein signaling 2	*Mus musculus*	O08849	41.176	0.048
Phypoly_transcript_02603	*ribA*	Poly(ADP-ribose) glycohydrolase	*Drosophila melanogaster*	O46043	42.67	4.66E-95
Phypoly_transcript_01309	*ribB*	Poly(ADP-ribose) glycohydrolase	*Oryza sativa Japonica Group*	Q9AV81	50.313	6.02E-146
Phypoly_transcript_06597	*spiA*	Polyribonucleotide nucleotidyltransferase	*Staphylococcus haemolyticus*	Q4L5X7	23.932	0.13
Phypoly_transcript_02655	*tspA*	Tumor suppressor p53-binding protein 1 homolog	*Caenorhabditis elegans*	Q7JKP6	33.803	1.16E-05
Phypoly_transcript_03260	*uchA*	Secretory immunoglobulin A-binding protein EsiB	*Escherichia coli*	A0A0H2VDN9	33.562	9.47E-14

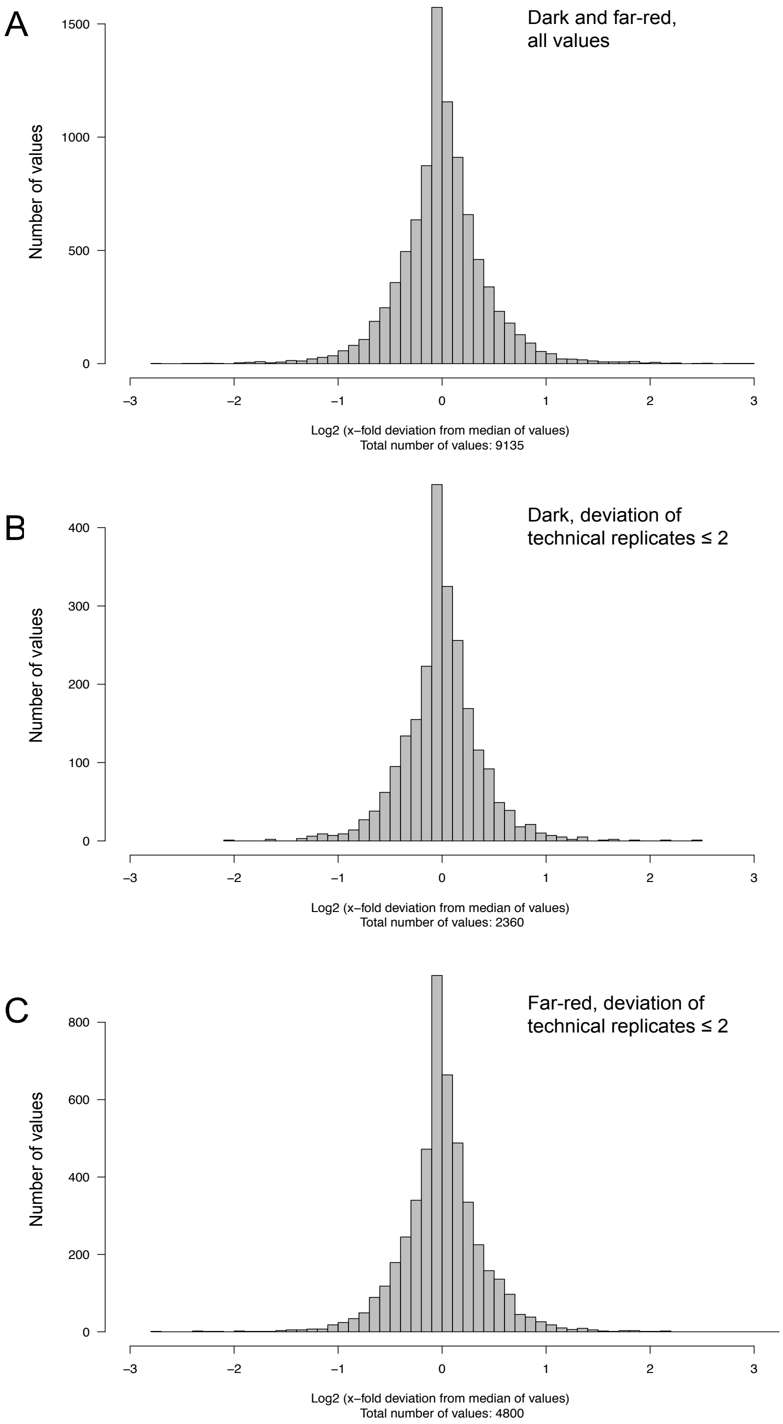

SI Figure 1. Homogeneity in gene expression, assayed by multiple sampling of individual plasmodial cells. In total, 43 plasmodia (15 dark controls and 28 far-red stimulated plasmodia, analysed at 6h after the stimulus pulse) were evaluated. Nine (3 x 3) or sixteen (4 x 4) samples were simultaneously taken from the same plasmodium and the concentration of the mRNAs of the set of 35 genes (SI Table 2) was determined twice by RT-PCR for each RNA sample. The frequency distributions display the Log2 of the x-fold deviation of each expression value from the median of all values for each gene. Panel (A) displays all values, measured in far-red stimulated cells and dark controls. Panels (B) and (C) display only those measurements where the two values of a RNA sample obtained by technical replication of the RT-PCR differed at maximum by a factor of two. Dark controls (B) and far-red stimulated cells (C) were evaluated separately

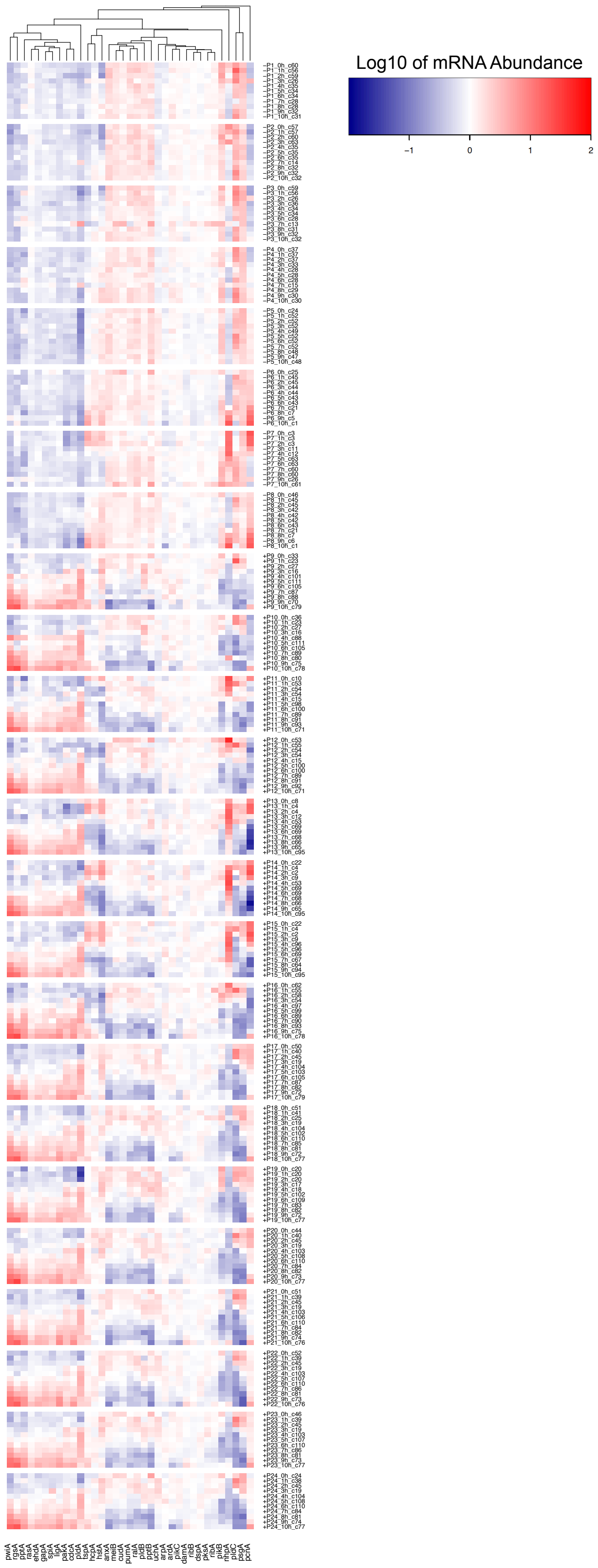

SI Figure 2. Heat map of time series of gene expression measured in individual plasmodial cells. Plasmodia are numbered according to Table 2. Each line of the heat map represents the gene expression pattern of a cell at the given time point. The label +P12_1h_c55, for example, indicates that the expression pattern of plasmodium number 12 as measured at 1h after the start of the experiment (corresponding to the onset of the far-red light stimulus in light-stimulated cells) was assigned to Simprof cluster number 55 and that the plasmodium had sporulated (+) in response to the stimulus (+, sporulated; -, not sporulated).

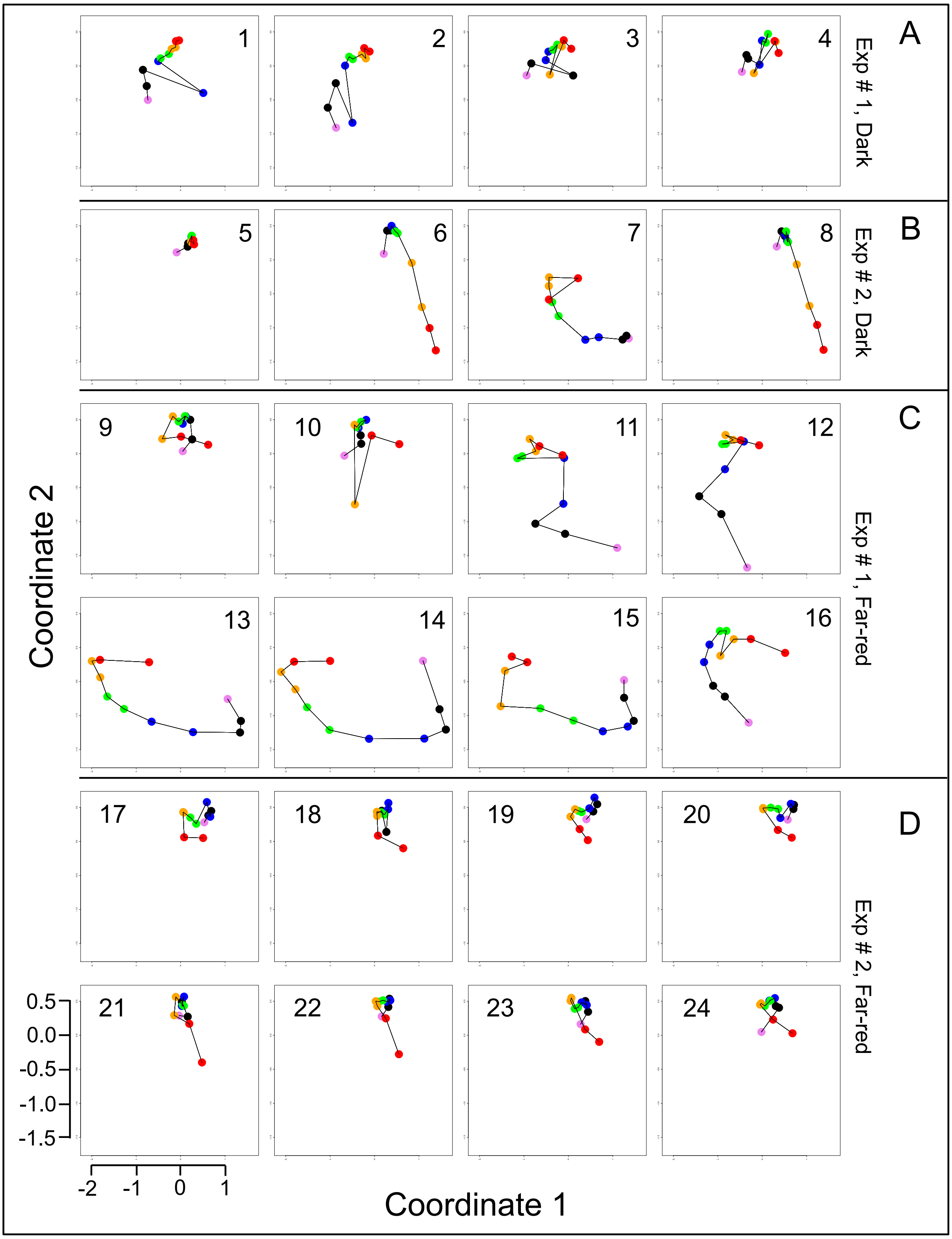

SI Figure 3. Single cell trajectories of gene expression displayed after multidimensional scaling (MDS) of the expression patterns of the *pcnA* group of genes (*hstA*, *nhpA*, *pcnA*, *uchA*). For further details see legend to Figure 3.

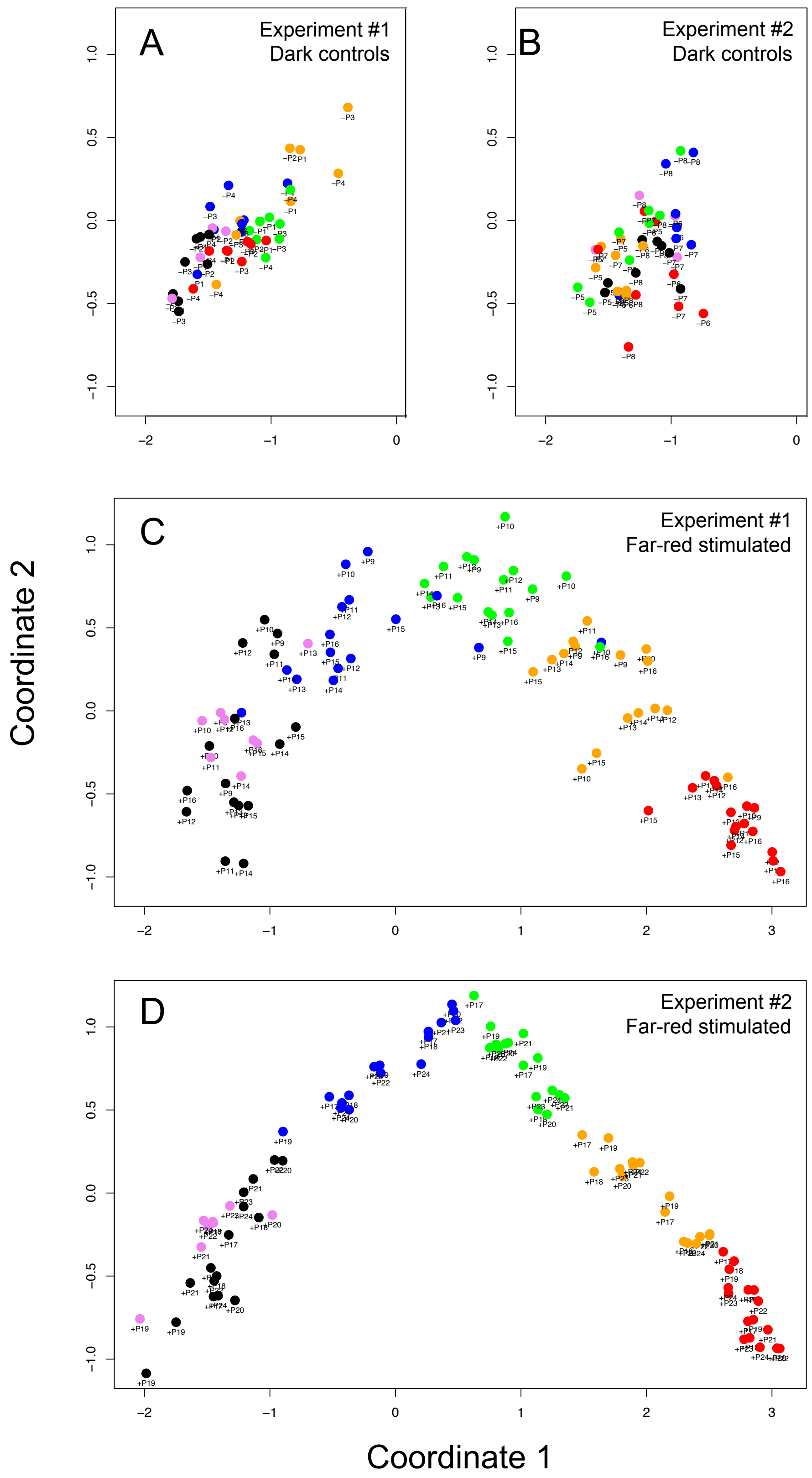

SI Figure 4. Multidimensional scaling of expression patterns of genes that were obviously up- (*cdcA*, *ehdA*, *gapA*, *ligA*, *pakA*, *pldA*, *pptA*, *pwiA*, *rgsA*, *spiA*) or down-regulated (*anxA*, *cudA*, *meiB*, *pikB*, *pldB*, *pldC*, *pptB*, *psgA*, *pumA*, *ralA*). Time is encoded by color (0h, pink; 1h, 2h, black; 3h, 4h, blue; 5h, 6h, green; 7h, 8h, ocher; 9h, 10h, red).

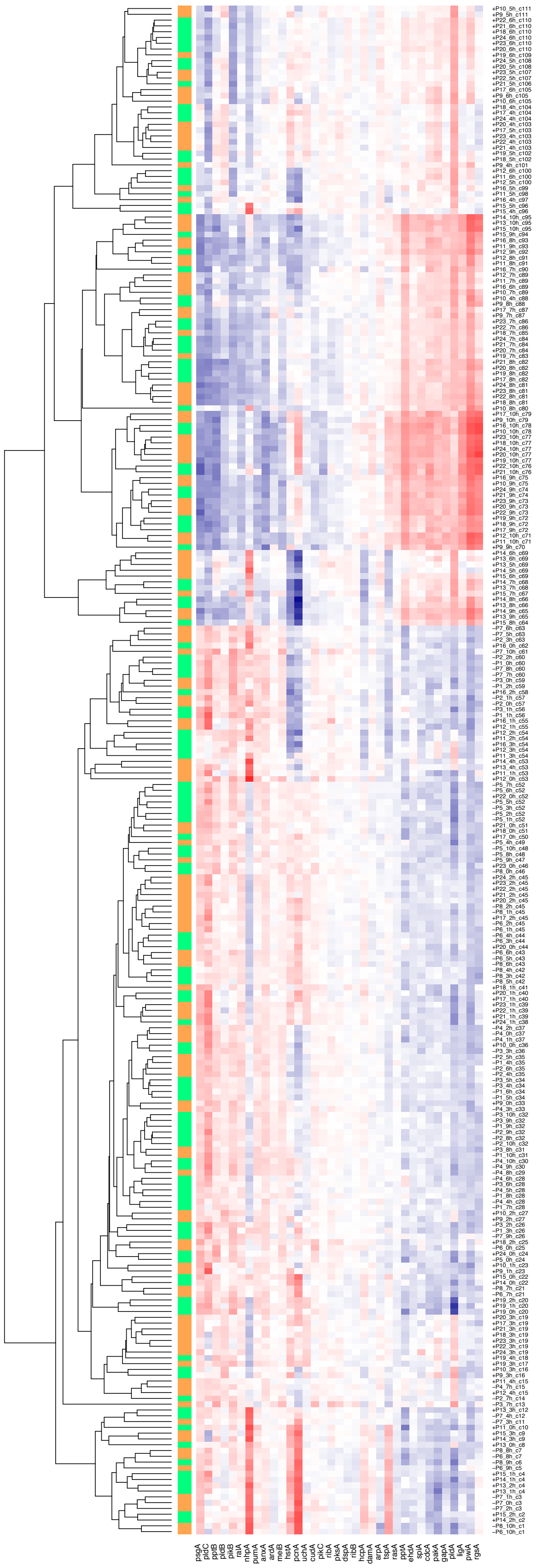

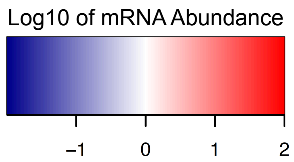

SI Figure 5. Heat map of gene expression values clustered by the Simprof algorithm. Each line of the heat map represents the gene expression pattern of a cell at a given time point. For labelling of expression patterns see legend to SI Figure 2. The side bar at the left side of the heat map indicates the borders of the individual clusters.

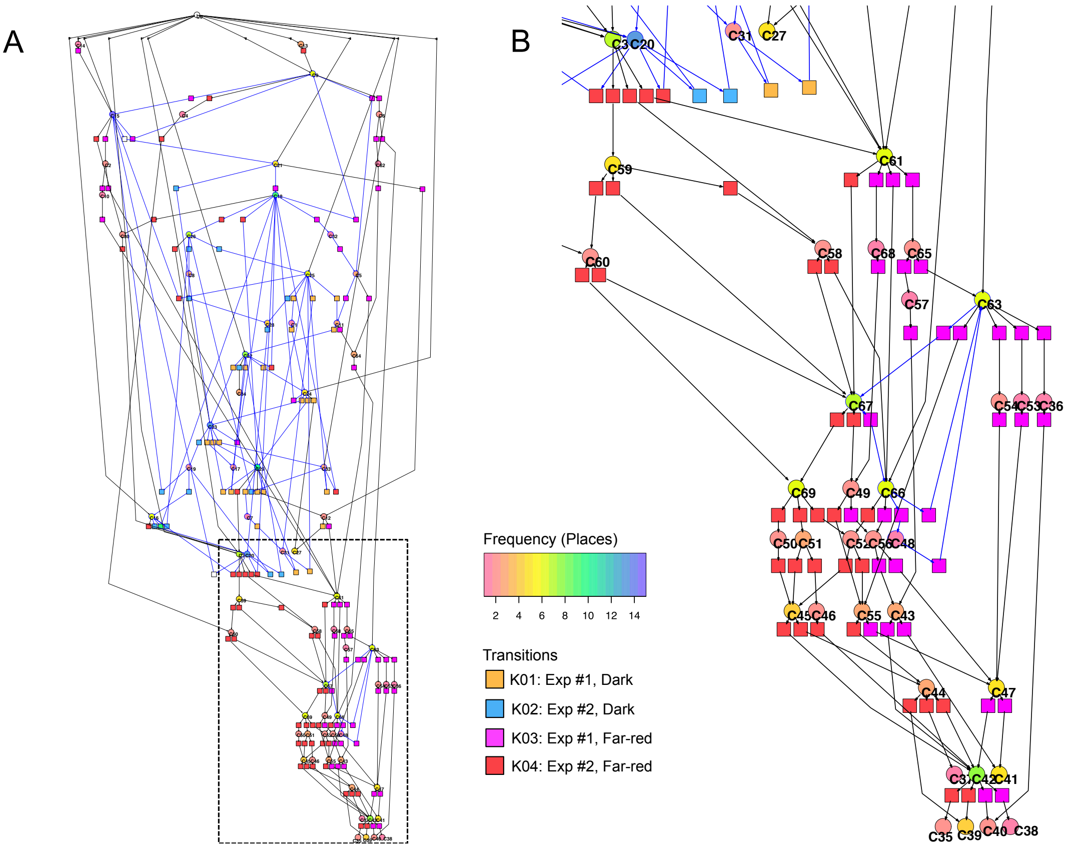

SI Figure 6. Petri net constructed for the set of up- or down-regulated genes, as listed in the legend to SI Figure 4. (A) Complete Petri net with a high number of T-invariants (indicated by arcs in blue) seen in the upper part of the net. (B) Magnified lower part of the Petri net in panel (A), marked by the rectangle of dashed lines. The part shown in (B) contains mainly transitions corresponding to far-red stimulated cells. Color coding of places indicates the relative frequencies of states of gene expression. Color coding of transitions indicates the group of cells (Table 2) in which the transits occurred.

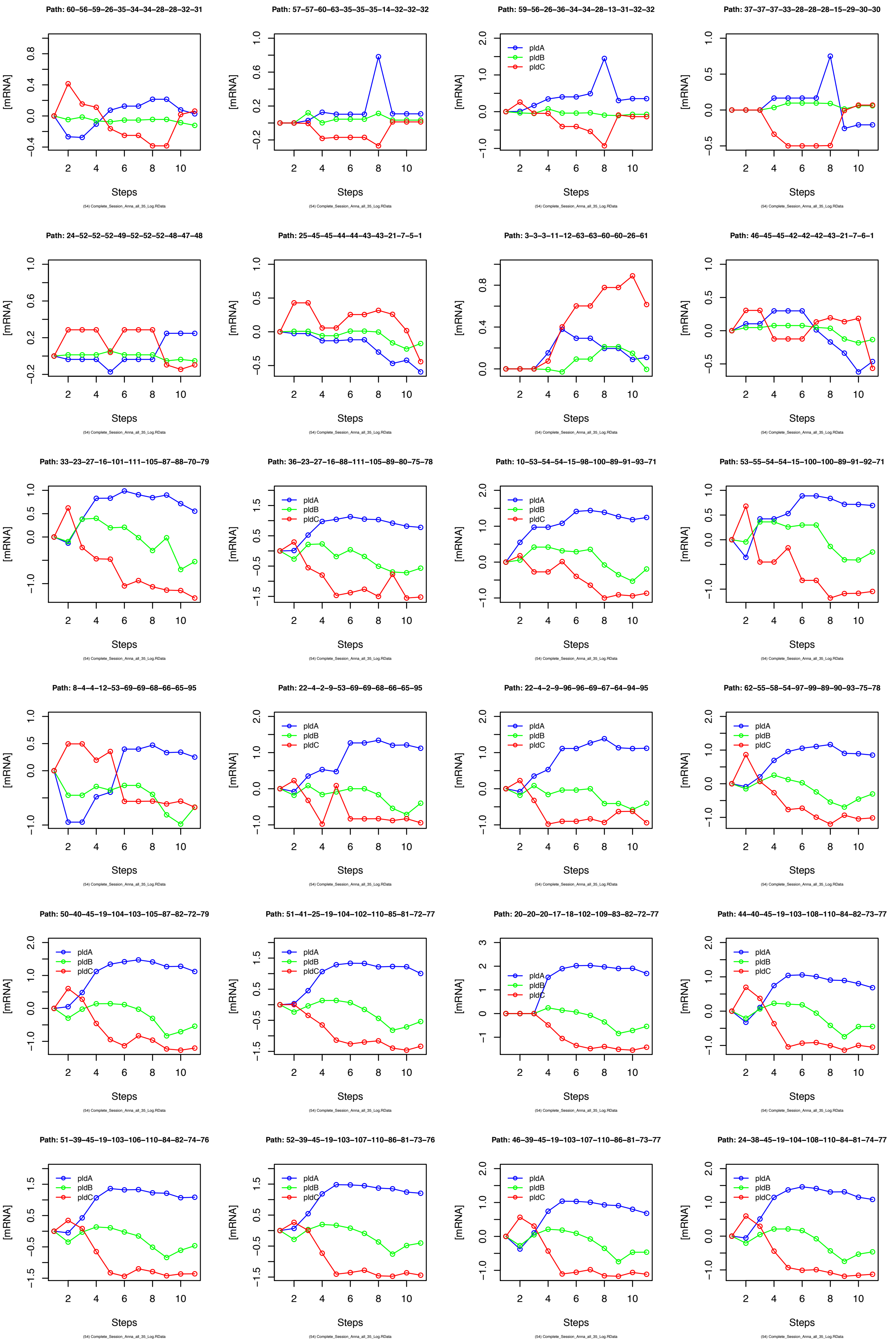

SI Figure 7. Gene expression kinetics as derived from single cell trajectories. For details see legend to Figure 9.

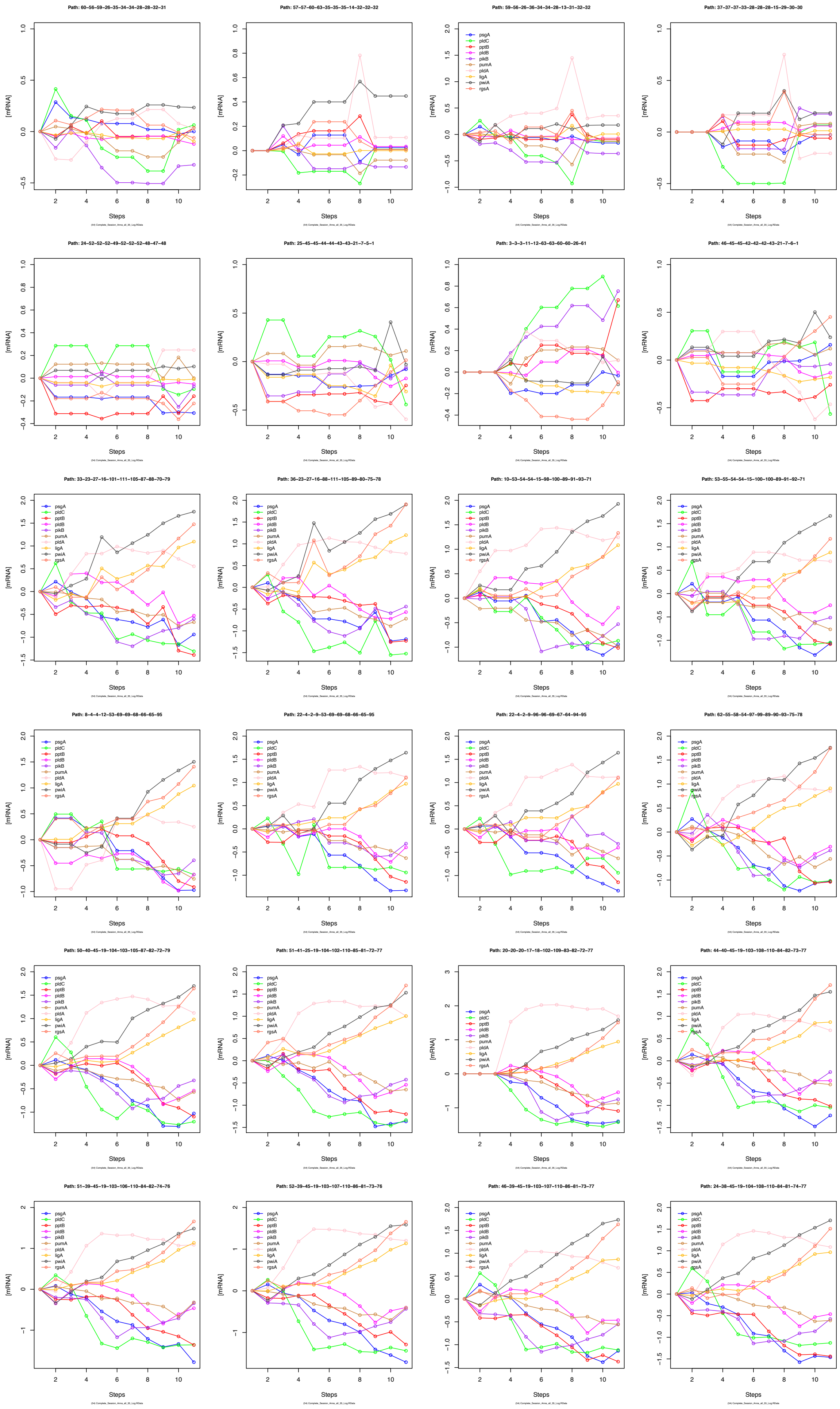

SI Figure 8. Gene expression kinetics as derived from single cell trajectories. For details see legend to Figure 9
